# Analysis of a rice blast resistance gene *Pita-Fuhui2663* and development of selection marker

**DOI:** 10.1038/s41598-022-19004-y

**Published:** 2022-09-01

**Authors:** Niqing He, Fenghuang Huang, Mingxiang Yu, Yebao Zhu, Qingshun Q. Li, Dewei Yang

**Affiliations:** 1 Rice Research Institute, Fujian High Quality Rice Research and Development Center, Fujian Academy of Agricultural Sciences, Fuzhou, 350019 Fujian China; 2 Biomedical Science Division, College of Dental Medicine, Western University of Health Sciences, Pomona, CA 91766 USA

**Keywords:** Genetics, Molecular biology

## Abstract

Rice blast is a detrimental rice disease caused by the fungus *Magnaporthe oryzae*. Here, we identified a resistance gene from the rice cultivar Fuhui 2663 which is resistant to the rice blast isolate KJ201. Through isolated population analyses and sequencing approaches, the candidate gene was traced to chromosome 12. With the use of a map-based cloning strategy, the resistance gene was ultimately mapped to an 80-kb resistance locus region containing the *Pita* gene. Candidate gene prediction and cDNA sequencing indicated that the target resistance gene in Fuhui 2663 was allelic to *Pita*, thus being referred to as *Pita-Fuhui2663* hereafter. Further analysis showed that the Fuhui 2663 protein had one amino acid change: Ala (A) residue 918 in Pita-Fuhui2663 was replaced by Ser (S) in Pita-S, leading to a significant change in the 3D structure of the Pita-S protein. CRISPR/Cas9 knockout experiments confirmed that *Pita-Fuhui2663* is responsible for the resistance phenotype of Fuhui 2663. Importantly, *Pita-Fuhui2663* did not affect the main agronomic traits of the variety compared to the *Pita* gene as verified by knockout experiments, indicative of potential applications of *Pita-Fuhui2663* in broader breeding programs. Furthermore, a *Pita-Fuhui2663-dCAPS* molecular marker with good specificity and high efficiency was developed to facilitate rice breeding for resistance to this devastating disease.

## Introduction

Rice (*Oryza sativa* L.) is one of the most important food crops worldwide. Rice blast, a fungal disease caused by the filamentous fungus *Magnaporthe oryzae* (synonym *Pyricularia oryzae*), is one of the most damaging diseases in rice production, leading to a significant yield reduction and considerably affecting the quality of rice^1,2^. The cloning and functional analysis of rice blast resistance genes and the study of *M. oryzae* effector proteins to explore the molecular mechanism of the interactions between rice and *M. oryzae* will increase the disease resistance of rice^3^. At present, more than 100 blast resistance genes have been identified, of which 37 have been cloned^4^. Most of these genes are dominant, and most of them, such as *Pita*, *Pi-1*, *Pi25, Pigm*, and *Pia*, encode resistance proteins. They can specifically recognize effectors and trigger a series of defense responses to inhibit the growth of pathogen, known as effector-triggered immunity (ETI)^1,5–8^. However, there are also disease-resistance genes that do not encode NBS-LRR. For example, *Pid2*, located on chromosome 6 and encoding a receptor-like kinase, is a constitutively expressed single-copy dominant gene^9^. *Pi21* encodes a protein with five proline-rich regions, and its resistance properties rely on the inactivation of protein function^10^. *Ptr* encodes an atypical broad-spectrum resistance protein containing four Armadillo repeat regions and may be a novel E3 ligase^11^.

A cluster of blast *R* genes on chromosome 12 of rice, including *Pita*, *Pita2*, and *Ptr*, has been effectively used in the breeding of blast resistant varieties worldwide^12,13^, suggesting that there may be signal recognition and transduction mechanisms among these *R* genes triggering rice immunity^15^. The *Pita* gene was cloned near the centromere on chromosome 12 of rice and encodes a 928-amino-acid cytoplasmic membrane receptor protein containing NBS-LRR domains^14^. *AVR-Pita* is the avirulent gene corresponding to *Pita*, which encodes a neutral zinc metalloprotease^15^. *Pita* was the first rice blast resistance protein confirmed to interact directly with an avirulent protein of the pathogen through its LRR domain, leading to resistance response^16^. Compared with *Pita*, *Pita2* shows a higher level and wider spectrum of disease resistance. All rice varieties containing *Pita2* have been reported to also contain *Pita*^14,17,18^. *Ptr* is a recently identified broad-spectrum rice blast resistance gene that is located physically close to the *Pita* region in the chromosome to the *Pita* region. *Ptr* may be specifically involved in *Pita*/*Pita2*-mediated resistance to rice blast and play a role in *Pita*-mediated signal recognition^16^. Further research has shown that *Ptr* is required for the function of *Pita*. The resistance mediated by *Pita* and *Pita2* was eradicated by the loss of *Ptr*, suggesting that the integrity of the *Ptr* gene product is key for pathogen signal transduction. *Ptr* was significant to in the broad-spectrum resistance of the *Pita* gene complex. Additionally, a *Ptr* mutant failed to recognize both *AVR-Pita* and *Pita2*-specific AVR genes, and all rice varieties carrying *Pita2* contained the same *Ptr* haplotype. It was preliminarily inferred that *Ptr* and *Pita2* may be the same gene, and *Ptr* was located about 210 kb upstream of *Pita*^11^. Recent research showed that *Ptr* and *Pita2* were alleles. The loss of function of *Pita2* but not *Pita* eliminated the specificity to some *AvrPita* containing isolates^19^.

*Pita* was the second blast resistance gene cloned in rice^14^. Due to close linkage with other resistance genes, *Pita*-containing varieties often have a broad blast resistance spectrum and thus have been popularized and well cultivated around the world^12,14,20,21^. The AVR-Pita_176_ protein binds directly to the Pi-ta LRD region inside the plant cell to initiate a Pi-ta-mediated defense response^9,19^. Intriguingly, a naturally occurring susceptible allele always encodes a protein that harbors a single amino acid substitution from alanine to serine at position 918 (A918S) of the Pita resistance protein^14,22^. The mutation of this residue has been shown to disrupt the interaction between Pita and its avirulent protein in a yeast system and an in vitro binding assay, indicating the importance of Ala at position 918 for the function of Pita^12^.

Here, we have identified a blast resistance gene in Fuhui 2663, an *Indica* rice cultivar resistant to blast fungus, and designated *Pita-Fuhui2663* was based on nucleotide polymorphism. *Pita-Fuhui2663* encodes an NBS-LRR protein containing an amino acid change, A918S, compared with the sequence of the Pita-S protein. Using CRISPR/Cas9 genome editing technology, we knocked out *Pita-Fuhui2663* in Fuhui 2663 and found that the knockout mutants lost its resistance, indicating that *Pita-Fuhui2663* confers blast resistance. Moreover, the analysis of knockout lines showed that the *Pita-Fuhui2663* gene did not affect the main agronomic traits of rice. We also developed a functional molecular marker, *Pita-Fuhui2663-dCAPS*, for *Pita-Fuhui2663*. These results suggest that *Pita-Fuhui2663* has good application prospects in rice blast resistance breeding.

## Materials and methods

### Statement

The current study complies with relevant institutional, national, and international guidelines and legislation for experimental research and field studies on plants (either cultivated or wild), including the collection of plant materials.

### Plant materials

The *Indica* rice CO39 and *Japonica* Lijiangxintuanheigu (LTH) were kept in the Rice Research Institute, Fujian Academy of Agricultural Sciences (Fuzhou, Fujian, China). Fuhui 2663, an *Indica* restorer line with good quality and blast resistance, was selected and bred by the Rice Research Institute. The F_1_ plants, generated from Minghui63 as female and Jiafuzhan as male, were back crossed with Minghui63 to produce the BC_1_F_1_ generation, and these lines were self-interbred for 9 generations, and a stable line was obtained, named as Fuhui 2663. Fuhui 2663 has been applied for a protected new national plant variety in 2021.

In the summer of 2019, Fuhui 2663 was used as a donor to cross with LTH and CO39. F_1_ seeds were sown in the spring and F_2_ seeds were harvested at the Sanya Experimental Station in Hainan Province, China. Fuhui 2663, and the two knockout lines (*Pita-Fuhui2663-KO-1* and *Pita-Fuhui2663-KO-2*) in the background of Fuhui 2663 were planted in the summer of 2021 at the Experimental Station in Fujian Province, China.

### *Magnaporthe oryzae* isolation

The *M. oryzae* isolate KJ201 was provided by the Fujian Academy of Agricultural Sciences. It was isolated by single-spore separation and stored on sterilized rice stems in a − 20 °C freezer.

### Inoculation and resistance evaluation of rice blast

The seeds were planted on petri dishes and, after 5 days, the uniformly sprouting seedlings were moved to small pots containing nutrient-rich soil. Each pot was planted with 12 seedlings, and three replicates were set up. The rice seedlings with 3–4 leaves were moved to an inoculation chamber with high humidity, and the spore suspension of the *M. oryzae* isolate KJ201 was sprayed on the plants with a spray gun. The inoculated plants were transferred to a plastic box covered with a wet sponge and cultured in a dark room (95–100% relative humidity (RH), 25 °C) for 24 h, and then transferred to a greenhouse at 25–28 °C for 6 days^23^. Grades 0–3 corresponded to resistant, and grades 4–9 corresponded to susceptible. The disease response was detected 1 week after inoculation, using susceptible cultivar CO39 or LTH as a control and the disease-resistant cultivar Fuhui 2663 as a positive control.

### Polymerase chain reaction amplification and marker detection

DNA was extracted from frozen leaves of rice by using the cetyltrimethylammonium bromide method^24^ with minor modifications. DNA polymerase chain reaction (PCR) amplification was performed as previously described^28^, amplification was performed using the following programme: 5 min at 94 °C; 35 cycles of 1 min at 94 °C, 40 s at 59 °C (for Indel) or 55 °C (for SSR) and 2 min at 72 °C; and a final extension of 5 min at 72 °C.

### Bulked segregant analysis

Bulked segregant analysis was used to search for markers linked to target genes. The leaf DNA of 20 susceptible plants randomly selected from the F_2_ population (Fuhui 2663 × LTH) was used to construct the susceptible DNA pool, and the leaf DNA of 20 resistant plants randomly selected from the F_2_ population (Fuhui 2663 × LTH) was used to construct the resistant DNA pool. SSR markers distributed in the rice genome were used for linkage detection, and DNAs extracted from LTH and Fuhui 2663 were used as control. The band type of the marker linked to the susceptible gene was the same as that of LTH.

### Primary mapping of the *Pita-Fuhui2663* gene

The localization population was constructed by crossing Fuhui 2663 (*Indica*) with LTH (*Japonica*). The preliminary mapping of *Pita-Fuhui2663* was randomly selected from 45 susceptible plants in all the F_2_ population (Fuhui 2663 × LTH), and a total of 536 susceptible plants were screened out for fine mapping in all the F_2_ population (Fuhui 2663 × LTH). MAPMAKER 3.0^25^ was used for linkage analysis of *Pita-Fuhui2663* loci and SSR markers, as reported by Rahman^26,27^.

### Fine mapping of the *Pita-Fuhui2663* gene

The bioinformatics analysis of the cultivar Nipponbare, published by the International Rice Genome Sequencing Project (IRGSP, http://rgp.dna.affrc.go.jp/IRGSP/index.html), was carried out to construct physical maps of target genes. The clones were anchored to the target gene linkage markers, and sequences were compared using BLAST (http://www.ncbi.nlm.nih.gov/blast/bl2seq/b12.html).

### Bioinformatics analysis

Candidate genes were predicted on the basis of existing sequence annotation databases (http://rice.plantbiology.msu.edu/; http://www.tigr.org/). Rice plant DNA and amino acid sequences were used for complete comparison using Clustal X version 1.81. The design of the endonuclease site was based on the available database (http://helix.wustl.edu/dcaps/). The spatial structures of the* Pita-Fuhui2663* and Pita-S proteins were predicted according to Robetta (https://robetta.bakerlab.org/submit.php).

### Sequencing of candidate genes

Primers were designed according to the predicted full-length sequence of candidate genes. Generally, one primer was designed according to the 3’UTR region of each candidate gene, and then another primer was designed according to the 5’UTR region. The Fuhui 2663 genes was sequenced according to the designed primers.

### Targeted mutagenesis of *Pita-Fuhui2663* with CRISPR/Cas9

The *Pita-Fuhui2663* gene in Fuhui 2663 was targeted with a gRNA spacer that spanned the two exons of the gene. The highly specific gRNA spacer sequences (Supplementary Table 1) were designed using the CRISPR-plant database and website^28^. Upon transformation, the regenerated plants were analyzed for genome editing mutations in target genes. Individual strains were selected from transgenic CRISPR-edited cell lines for the sequencing of specific mutations in PCR products^29^. About 300 rice seeds of Fuhui 2663 were subjected to mutation, about 150 seeds were recovered after antibiotic screening, about 20 positive seedlings were obtained by PCR after rooting, and then 5 mutants were obtained by sequencing, and finally 2 stable independent homozygous knockout lines (*Pita-Fuhui2663-KO-1* and *Pita-Fuhui2663-KO-2*) were obtained. The primers for CRISPR/Cas9 used in this study are shown in Supplementary Table 1.

### Declarations

The current study complies with relevant institutional, national, and international guidelines and legislation for experimental research and field studies on plants (either cultivated or wild), including the collection of plant materials.

## Results

### Genetic analysis of the resistance gene

**Figure 1:**
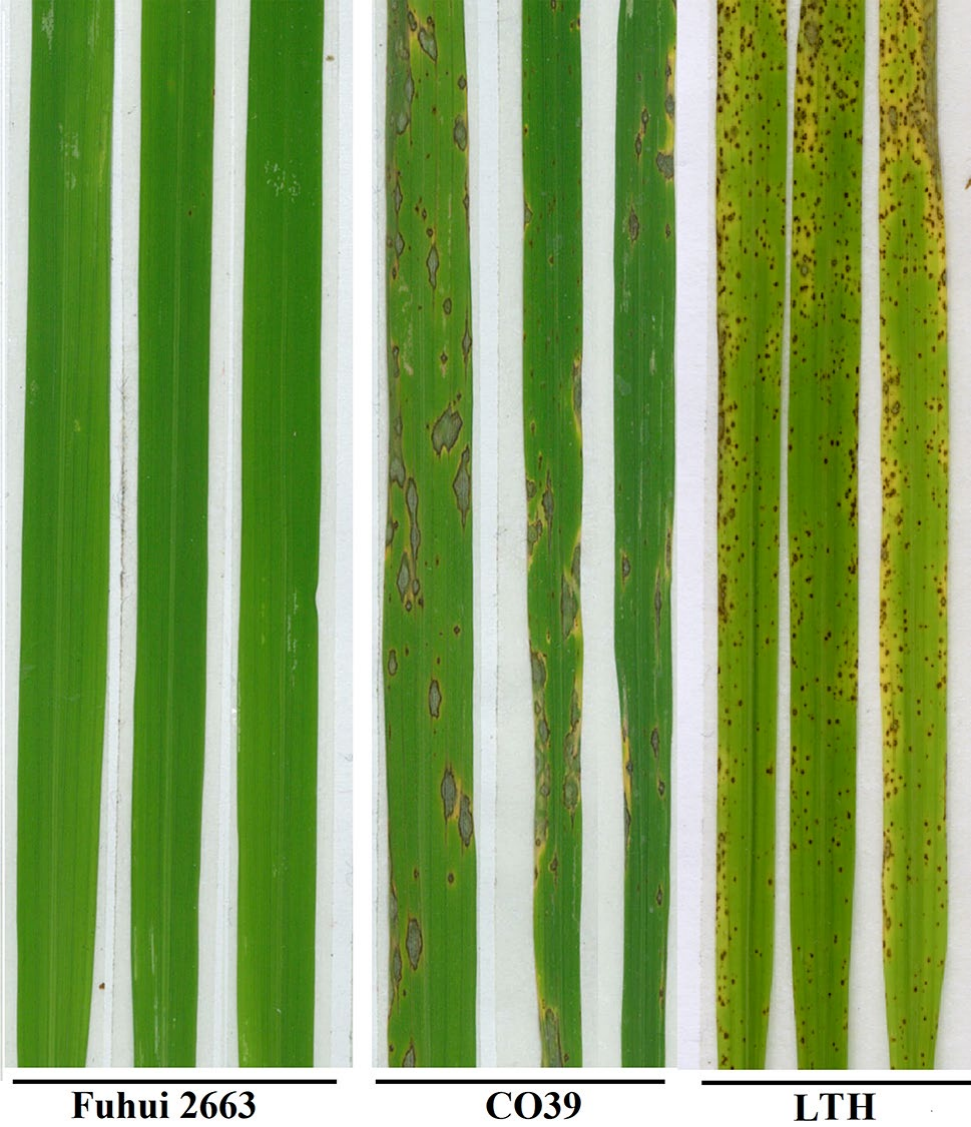
Fuhui 2663 is resistant to KJ201 under laboratory conditions. Plants of Fuhui 2663 and CO39 at the 3–4-leaf stage were transferred to an inoculation chamber under high humidity, and 25 mL of spore suspension of *M. oryzae* isolate KJ201 was sprayed with an air compressor. The inoculated plants were transferred to a plastic box covered with a wet sponge, cultured in a dark room (95–100% RH, 25 °C) for 24 h, and then transferred to a greenhouse at 25–28 °C for 6 days, after which photographs were taken.

To analyze the blast resistance of Fuhui 2663 in the laboratory, 2-week-old plants growing in the greenhouse were inoculated with blast fungus KJ201 isolate. Using susceptible rice CO39 and LTH as controls, Fuhui 2663 was found to have strong resistance to blast KJ201 (Fig. 1 and Supplementary Fig. 1).

For genetic analysis of Fuhui 2663, the resistance donor Fuhui 2663 was crossbred with susceptible parents CO39 and LTH. A total of 36 F_1_ individuals (the Fuhui 2663 × CO39 and the Fuhui 2663 × LTH) showed a resistance phenotype against the *M. oryzae* isolate KJ201. The segregation of resistant (*R*) and susceptible (*S*) progenies in the F_2_ population fitted a 3:1 ratio (the Fuhui 2663 × CO39 F_2_ population, 412 R: 139 S, v2 = 0.305; the Fuhui 2663 × LTH F_2_ population, 382 R:124 S, v2 = 0.34, Table 1). The *R/S* ratio showed that Fuhui 2663 contained a dominant resistance gene.

**Table 1:** Resistance of Fuhui 2663 and hybrid offspring to the rice blast isolate KJ201.

Cross	F_1_ phenotype	F_2_ population			χ^2^ (3:1)	*P*
		Resistant individuals	Susceptible individuals	Total plants		
Fuhui 2663 × CO39	Disease resistant	412	139	551	0.305*	0.5–0.75
Fuhui 2663 × LTH	Disease resistant	382	124	409	0.34*	> 0.9

*Denotes that the segregation ratio of resistant plants to susceptible plants complied with 3:1 at the 0.05 significance level.

### Mapping of the resistance gene using molecular markers

To further determine the gene responsible for the resistance phenotype of Fuhui 2663, 506 SSR markers were obtained from the rice molecular map, and 317 primer pairs of markers showed polymorphisms between Fuhui 2663 and CO39. These 317 pairs of primer markers were used for linkage analysis of the DNA pools from 20 resistant plants or 20 susceptible plants in the F_2_ population (Fuhui 2663 × LTH). Each primer pair was used for the detection of 4 DNA samples (Fuhui 2663, CO39, the pool of 20 resistant plants, and the pool of 20 susceptible plants). When using indel-12-4 and indel-12-7 as primers, the test results showed that the size of the PCR product of Fuhui 2663 was the same as that of the DNA pool of 20 resistant plants, while CO39 was the same as that of the DNA pool from 20 susceptible plants. Therefore, we speculated that the Indel-12-4 and Indel-12-7 markers may be linked to the resistance gene.

**Figure 2:**
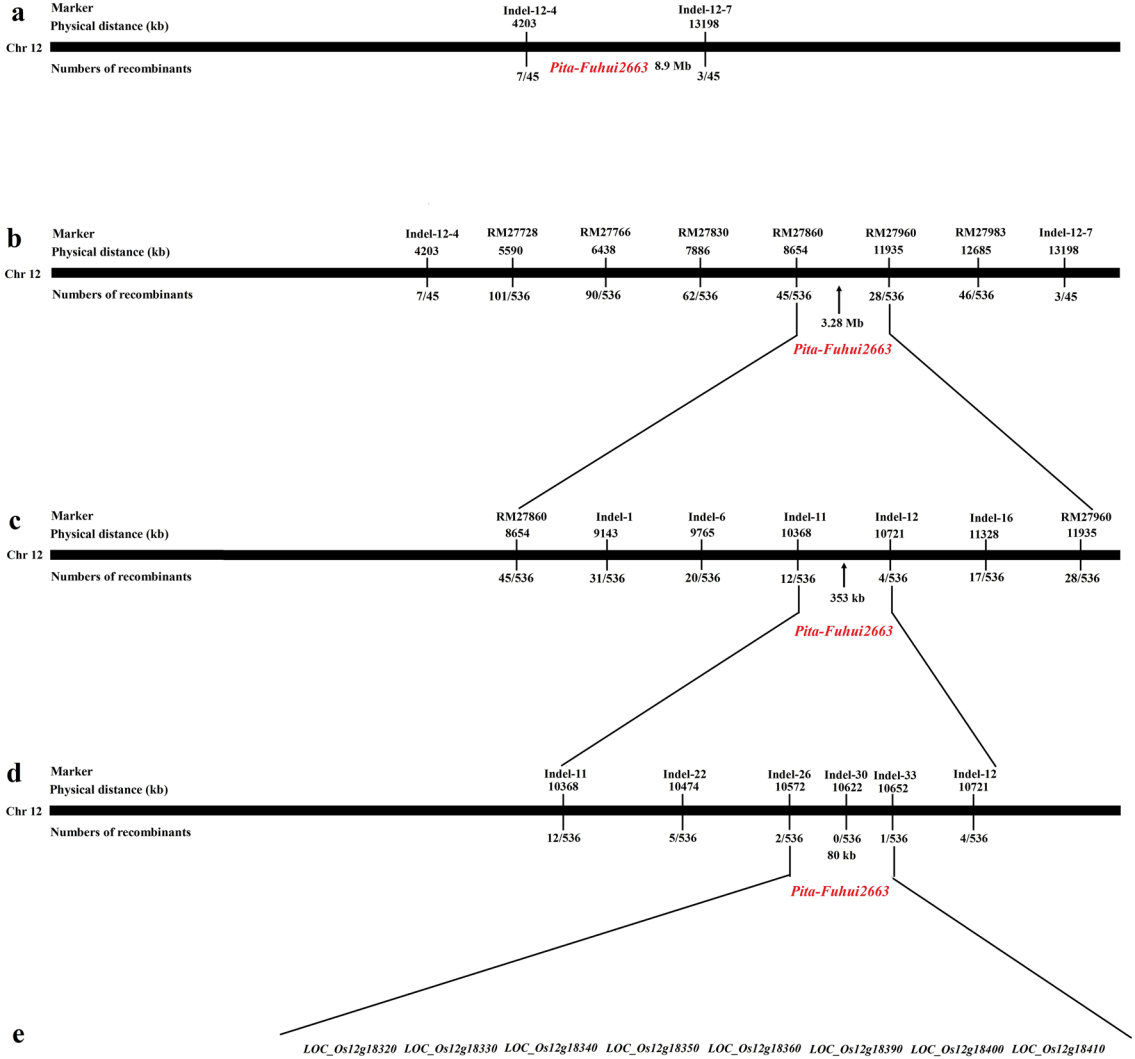
Genetic and physical maps of the *Pita-Fuhui2663* gene. (**a**) Primary mapping of the resistance gene; the *Pita-Fuhui2663* gene was identified between markers Indel-12-4 and Indel-12-7. (**b**) Further mapping of the resistance gene; the *Pita-Fuhui2663* gene was identified between markers RM27860 and RM27960. (**c**) Fine mapping of the resistance gene; the *Pita-Fuhui2663* gene was identified between markers Indel-11 and Indel-12. (**d**) High-resolution mapping of the resistance gene; the *Pita-Fuhui2663* gene was eventually mapped to an 80-kb region between markers Indel-26 and Indel-33, and the number of recombinations between the marker and the target gene is shown on the linkage map. (**e**) There were eight annotated genes in the 80-kb region.

To preliminarily locate this resistance gene, 45 susceptible individual plants were used for further validation, and the results showed that Indel-12-4 and Indel-12-7 were linked to the resistance locus. Therefore, the resistance gene was located between the markers Indel-12-4 and Indel-12-7 on chromosome 12, with an estimated physical distance of about 8.9 Mb (Fig. 2a).

### Fine mapping of the resistance gene

To map the resistance gene to a smaller region, 536 susceptible individual plants were identified from the F_2_ population (Fuhui 2663 × LTH). Further mapping was performed by using published markers (http://archive.gramene.org/markers/), and six SSR polymorphisms (RM27728, RM27766, RM27830, RM27860, RM27960 and RM27983) were selected from 18 markers between Indel-12-4 and Indel-12-7. Using these six markers, the resistance gene was found to be located between the molecular markers RM27860 and RM27960 with a physical distance of 3.28 Mb (Fig. 2b and Supplementary Table 2).

To further localize the resistance gene, five Indel polymorphisms were selected from 20 new markers between RM27860 and RM27960. Indel marker development referred to the insertion or deletion of nucleotide fragments of different sizes at the same site of the genome between related species or different individuals of the same species, and Indel markers were designed on the basis of published rice genome sequences, and the polymorphisms between Fuhui 2663 and CO39 were predicted by comparing the sequences of Nipponbare (http://rgp.dna.affrc.go.jp/) and 93–11 (http://rice.genomics.org.cn/). The resistance gene was found to be located between the molecular markers Indel-11 and Indel-12 on chromosome 12, and the physical distance between the two markers was 353 kb (Fig. 2c and Supplementary Table 2).

For further fine mapping, four polymorphic Indels were selected from 15 new Indel markers (Supplementary Table 2). Six markers (Indel-11, Indel-22, Indel-26, Indel-30, Indel-33, and Indel-12) were used for recombinant screening, and 12, 5, 2, 0, 1, and four recombinant plants were detected, respectively. Therefore, the resistance gene was precisely located in the estimated 80 kb region between Indel-26 and Indel-33 (Fig. 2d).

### Candidate genes in the 80 kb region

According to the existing sequence annotation databases (http://rice.plantbiology.msu.edu/; http://www.tigr.org/), there were eight candidate genes in the 80 kb region identified (Fig. 2e), and all genes had a corresponding full-length cDNA. LOC_Os12g18320*,* LOC_Os12g18330, LOC_Os12g18340, LOC_Os12g18350 and LOC_Os12g18400 encode retrotransposon proteins; LOC_Os12g18360 encodes an NBS-LRR protein; LOC_Os12g18390 encodes a centromere-specific protein; LOC_Os12g18400 encodes a mitochondrial iron-regulated protein (MIR); and LOC_Os12g18410 encodes a retrotransposon protein and also the MIR protein.

### Sequence analyses of the resistance gene

**Figure 3:**
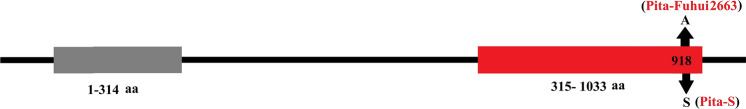
Structure comparison between* Pita-Fuhui2663* and Pita-S. DNA sequencing analysis showed that there was only one amino acid substitution (A918S) between* Pita-Fuhui2663* and Pita-S.

Further analysis revealed that LOC_Os12g18360, encoding an NBS-LRR protein, was the *Pita* gene in this locus^14^. To see if *Pita* was involved in the resistance phenotypes, we next sequenced *Pita* in Fuhui 2663 and CO39. Sequencing revealed a base substitution that resulted in the mutation of amino acid 918 from alanine (A) to serine (S) (Fig. 3), we speculated that this mutation led to the loss of resistance. These results indicated that this resistance gene in Fuhui 2663 was most likely allelic to *Pita.* Therefore, we named this gene *Pita-Fuhui2663.* According to the position of *Ptr, Ptr* was located about 210 kb upstream of *Pita-Fuhui2663* and *Pita* (Supplementary Fig. 2)*.*

**Figure 4:**
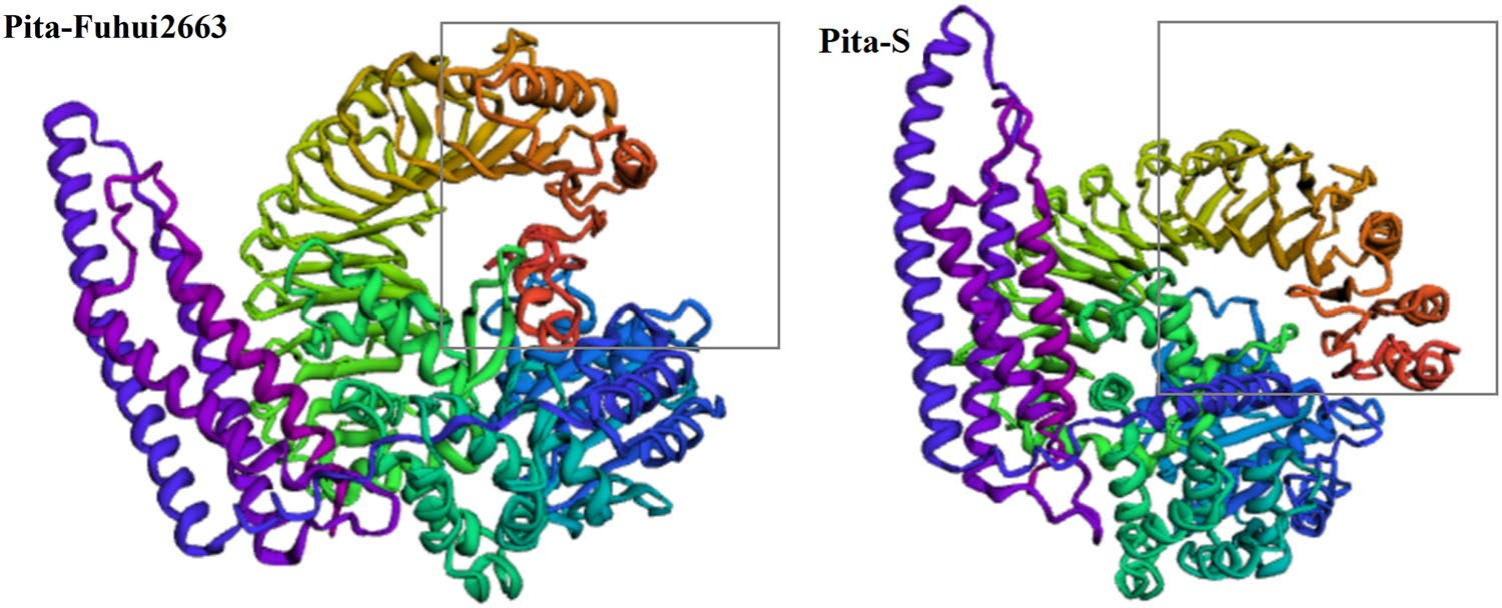
Spatial structures of the Pita-Fuhui2663 and Pita-S proteins. There are significant structural changes in the LRR domain based on the Robettamodel program, and the 918th amino acid is substituted from alanine (Pita-Fuhui2663) to serine (Pita-S), which is likely to affect Pita-S protein function. The gray square indicates the site of structural changes.

Because alanine is a nonpolar amino acid and serine is a polar amino acid, this substitution can be expected to alter the structure of the Pita-S protein. By simulating the spatial structure of* Pita-Fuhui2663* and Pita-S based on the Robettamodel program, we observed a structural change between the* Pita-Fuhui2663* and Pita-S proteins in the LRR domain (Fig. 4), speculating that Ala_918_ is an important site for* Pita-Fuhui2663*, and the mutation to Ser_918_ may partly affect the function of the Pita-S protein.

### *Pita-Fuhui2663* is responsible for the resistance of Fuhui 2663 to blast

**Figure 5:**
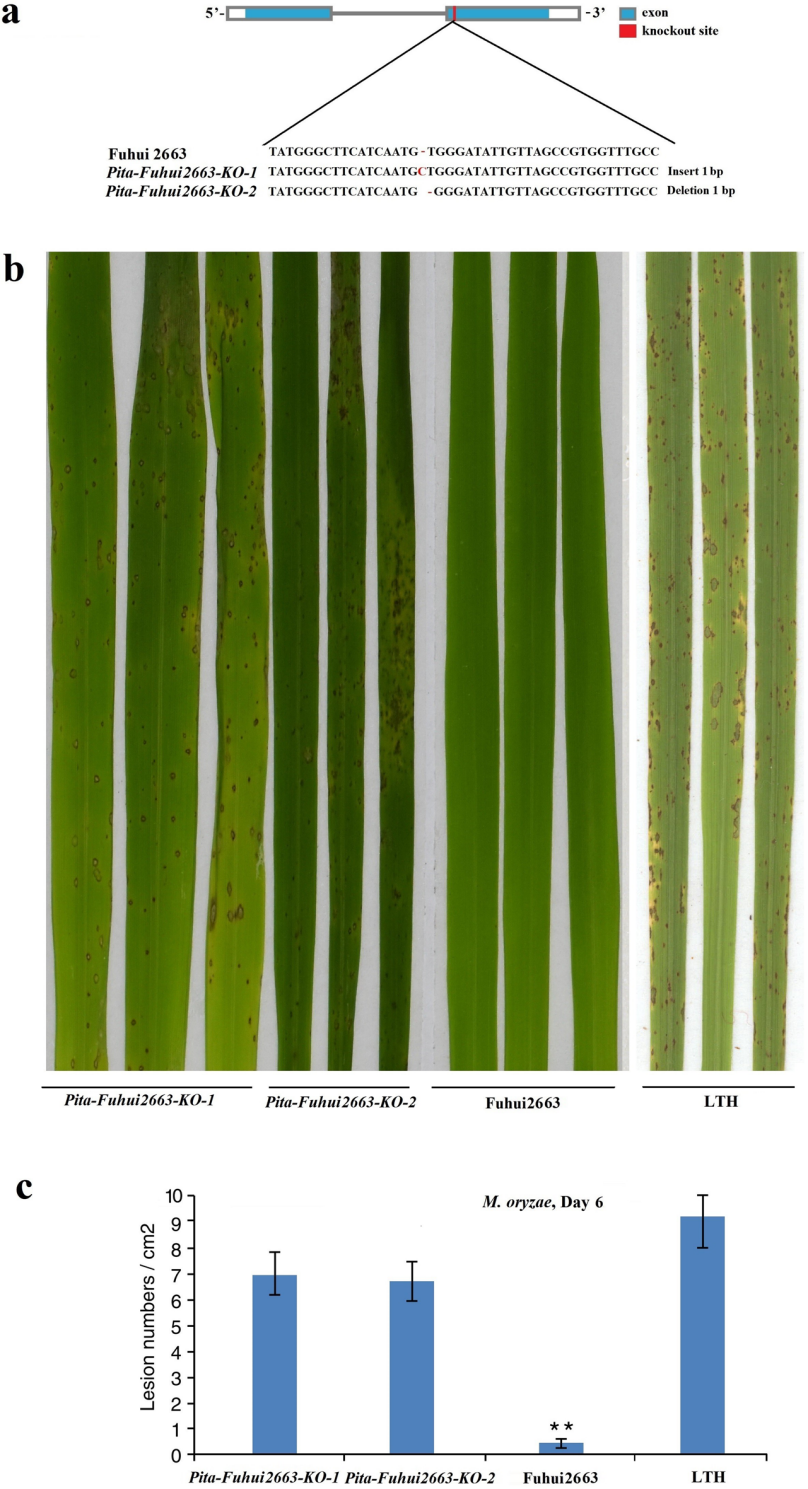
*Pita-Fuhui2663* knockout lines showed enhanced susceptibility to KJ201. (**a**) Two independent lines (designated *Pita-Fuhui2663-KO-1* and *Pita-Fuhui2663-KO-2*) were generated using the CRISPR/Cas9 system and verified by sequencing. (**b**) Inoculation of rice blast fungus showed that the two knockout lines generated by CRISPR/Cas9 were susceptible to KJ201, while their parent Fuhui2663 was resistant to KJ201. Leaves were photographed 6 days post-infection with *M. oryzae* isolate KJ201. (**c**) Lesion numbers per cm^2^ on the rice leaves (M ± SD, n > 6 leaves) after inoculation with blast fungus as in (**b**). **denotes *P* < 0.01.

To confirm that *Pita-Fuhui2663* is responsible for blast resistance, we investigated whether gene knockout of *Pita-Fuhui2663* in the resistant cultivar Fuhui 2663 resulted in a susceptible phenotype. To this end, a sequence-specific guide RNA (gRNA) for the second exon of *Pita-Fuhui2663* was designed using the CRISPR/Cas9 gene-editing system. Two plants (*Pita-Fuhui2663-KO-1* and *Pita-Fuhui2663-KO-2*) were obtained from two independent events and were found to have a 1 bp insertion or deletion within a targeted site based on sequencing (Fig. 5a, Supplementary Table 3), affect their amino acid sequence changes (Supplementary Fig. 3).

We then inoculated the two homozygous mutants with KJ201, and both lines were completely susceptible to KJ201 (Fig. 5b,c). Therefore, we concluded that the targeted mutation of *Pita-Fuhui2663* in the resistance of Fuhui 2663 led to disease susceptibility to KJ201, demonstrating that *Pita-Fuhui2663* is responsible for blast resistance in Fuhui 2663.

### Main agronomic characteristics of Fuhui 2663 and the two knockout lines

We next monitored several key agronomic traits in Fuhui 2663 and the two knockout lines (*Pita-Fuhui2663-KO-1* and *Pita-Fuhui2663-KO-2*) to assess their potential in rice breeding applications. We compared yield-related traits, including plant height, panicle length, number of effective panicles, spikelets per panicle, seed setting rate, thousand-grain weight, and grain length and width. The results showed that there was no significant difference in these main agronomic characteristics between Fuhui 2663 and the two knockout lines (Table 2), suggesting that *Pita-Fuhui2663* mainly played a role in rice blast resistance and did not affect other agronomic traits, likely having good breeding value and potential in the future.

**Table 2:** Comparison of the main agronomical traits of Fuhui 2663 and the *Pita-Fuhui2663* knockout lines.

Trait	Fuhui 2663	*Pita-Fuhui2663-KO-1*	*Pita-Fuhui2663-KO-2*
Plant height (cm)	122.32 ± 2.86	121.65 ± 3.02	122.12 ± 3.13
Panicle length (cm)	24.06 ± 1.22	23.96 ± 1.34	23.88 ± 1.62
Number of effective panicles	9.98 ± 1.12	10.01 ± 1.02	10.12 ± 1.04
Spikelets per panicle	168.68 ± 4.32	169.68 ± 5.14	168.68 ± 4.88
Seed setting rate (%)	94.12 ± 1.36	92.18 ± 2.12	92.38 ± 2.14
1,000-grain weight (g)	26.46 ± 0.58	27.12 ± 0.61	26.88 ± 0.52
Grain length (mm)	11.42 ± 0.13	11.60 ± 0.19	11.66 ± 0.22
Grain width (mm)	2.42 ± 0.06	2.45 ± 0.07	2.41 ± 0.09

### Development of a functional marker for the *Pita-Fuhui2663* gene

Considering the importance of the *Pita-Fuhui2663* gene and the deficiency of existing markers, we developed a *Pita-Fuhui2663-dCAPS* molecular marker using cloned *Pita-Fuhui2663* cDNA sequences for efficient marker-assisted selection (Supplementary Table 1). There is a single base substitution between *Pita-Fuhui2663* and *Pita-S*. According to this difference, we found an *Eco*P15I enzyme digestion site. The susceptible gene *Pita-S* could be specifically recognized by the endonuclease *Eco*P15I, and a 98 bp fragment could be amplified, and Lane 2 showed the amplified target fragment of 98 bp from *Pita-S*, while the resistance *Pita-Fuhui2663* could not, and could only be amplified to a 123 bp fragment, where Lane 1 showed the amplified target fragment of 123 bp from *Pita-Fuhui2663* (Supplementary Fig. 4).

## Discussion

### Genetic and functional analysis of the *Pita-Fuhui2663* gene

Although *Pita* had been cloned, how this residue contributes to the association between Pita and AVR-Pita is unclear. Here, we simulated the spatial structure of* Pita-Fuhui2663* and its susceptible variant Pita-S (A918S) and found a structural change in the LRR domain between Pita-Fuhui2663 and Pita-S. In addition, the two knockout mutants also showed different protein structural changes (Fig. 4). Considering the polarity difference between these two amino acids, Pita-S might show a change in the hydrophobic environment provided by Ala, hindering protein interactions. Meanwhile, abundant blast *R* genes are present not only within but also between species. Within the same rapidly evolving gene family, *R* genes can exhibit an effector response to develop resistance to rapidly evolving fungal pathogens. This was included in a proposed unique mechanism called "restricted differentiation", in which *R* genes and pathogen effectors can only follow a limited evolutionary path to improve fitness^30^. However, further research should be conducted to uncover the molecular machinery.

### Analysis of the application prospects of the *Pita-Fuhui2663* gene

Fuhui 2663 is a new restorer line bred by our team that harbors excellent characteristics for the paddy field, with a good leaf shape, high seed setting rate, and considerable resistance to blast disease. In this study, we used fine mapping to determine *Pita-Fuhui2663* as the single dominant gene that confers Fuhui 2663 blast resistance and performed the functional identification of this gene through the use of the CRISPR/Cas9 system. Previously, a subset of rice core accessions in the United States was collected to evaluate the relationship between blast resistance and yield-related components, revealing that rice genomes with *Pita* correlated with lighter seed weights^31^. Recent studies have shown that susceptible *Pita* has an important effect on rice yield, and different *Pita* allelic mutants have different effects on rice yield^32^. However, our data showed that, except for disease susceptibility, the two *Pita-Fuhui2663* knockout lines based on Fuhui 2663 showed no significant differences in other agronomic traits, including thousand-grain weight. These suggest that the presence of *Pita-Fuhui2663* has little impact on the growth and development of Fuhui 2663. We speculate that one or more unknown factors may be expressed specifically in the genome of Fuhui 2663 to balance its defense and growth. Such a synergistic mechanism has been confirmed in some other blast resistance proteins. For instance, PigmR dimerization for resistance was attenuated competitively by its homolog PigmS, resulting in the suppression of immune responses and yield cost^1^. Therefore, elucidating the association between *Pita-Fuhui2663*-induced immunity and seed production in Fuhui 2663 warrants further studies.

In order to further analyze the distribution of *Pita-Fuhui2663*, we performed SNP (Single nucleotide polymorphisms) calling and haplotype (Hap) analysis of the 3000 sequenced rice genomes available in the CNCGB and CAAS databases^33^ and found 25 Haps for the *Pita-Fuhui2663* gene, including 9 Haps among more than 15 rice resource materials (Supplementary Table 4). Further analysis revealed that Hap 1 and Hap 9, a total of 703 materials contained *Pita-Fuhui2663*, while the other Haps were different from *Pita-Fuhui2663.* Therefore, of the 3,000 sequenced rice genomes, there were relatively few Haps containing *Pita-Fuhui2663.*

Marker-assisted selection is an important method to increase the efficiency of rice resistance breeding^34^. Jia et al.^35^ initially analyzed natural variation at the *Pita* locus and developed a set of dominant markers based on a PCR method. Afterward, Wang et al.^36^ further optimized the *Pita* marker system to two pairs of dominant markers, according to a polymorphic site in the intron (GCC to CTAT), which have been widely used in rice breeding. Here, we used the dCAPS method to develop a *Pita-Fuhui2663* functional marker using the restriction endonuclease *Eco*P15I. This marker depends on the crucial polymorphic site (A918S in proteins) in the second exon of *Pita-Fuhui2663*. It was accordingly used to identify the *Pita-Fuhui2663* resistance locus in our tests as a complement to the *Pita-Fuhui2663* marker system. Thus, to breed a new hybrid rice variety, breeders can transfer *Pita-Fuhui2663* into both restorer and sterile lines using molecular marker-assisted selection.

In conclusion, Fuhui 2663, with excellent comprehensive traits, contains the *Pita-Fuhui2663* resistance gene, and we believe that this rice variety will have great potential in future scientific research and breeding applications.

## Conclusions

In this study, using a map-based cloning strategy, the *Pita-Fuhui2663* gene from rice cultivar Fuhui 2663 was mapped to an 80-kb resistance locus region that contained the *Pita* gene. Then, CRISPR/Cas9 knockout experiments confirmed that *Pita-Fuhui2663* is responsible for the resistance phenotype of Fuhui 2663. Importantly, *Pita-Fuhui2663* did not affect the main agronomic traits of the variety compared to the *Pita* gene as verified by knockout experiments, indicative of potential applications of *Pita-Fuhui2663* in broader breeding programs. Finally, a *Pita-Fuhui2663-*dCAPS molecular marker with good specificity and high efficiency was developed, the application of which will facilitate combating this devastating disease.

## Supplementary Information

Supplementary Information.

## Data Availability

All data is included in this publication as figures, tables and supplements.
