# Correction: Analysis of a rice blast resistance gene *Pita-Fuhui2663* and development of selection marker

**DOI:** 10.1038/s41598-025-28622-1

**Published:** 2025-11-17

**Authors:** Niqing He, Fenghuang Huang, Mingxiang Yu, Yebao Zhu, Qingshun Q. Li, Dewei Yang

**Affiliations:** 1 Rice Research Institute, Fujian High Quality Rice Research and Development Center, Fujian Academy of Agricultural Sciences, Fuzhou, 350019 Fujian China; 2 Biomedical Science Division, College of Dental Medicine, Western University of Health Sciences, Pomona, CA 91766 USA

Correction to: *Scientific Reports* 10.1038/s41598-022-19004-y, published online 01 September 2022

The original version of this Article contained errors in Fig. 5, where an incorrect phenotypic type of the test series was shown. The original Fig. 5 appears below.

**Fig. 5:**
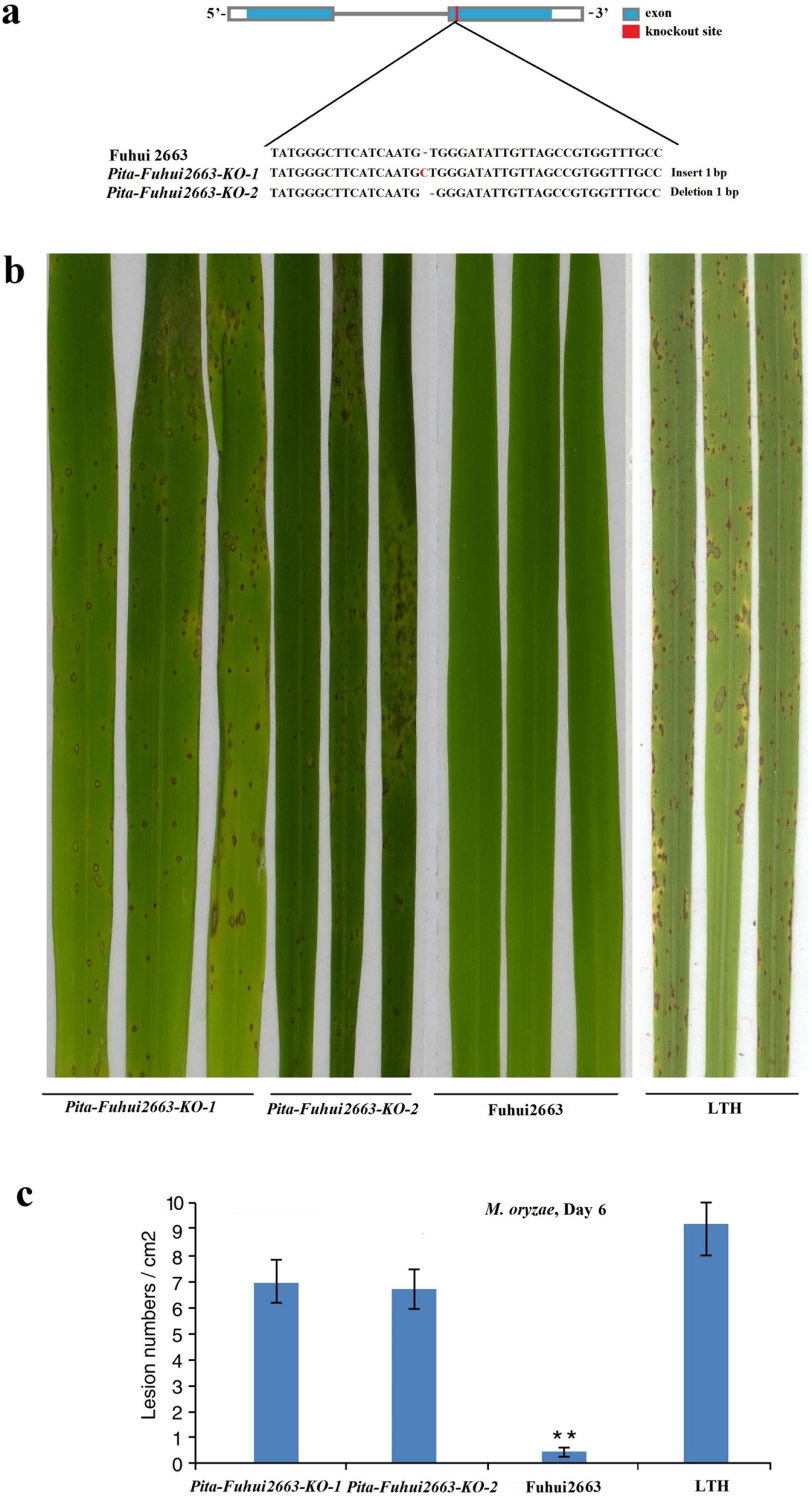
*Pita-Fuhui2663* knockout lines showed enhanced susceptibility to KJ201. (**a**) Two independent lines (designated *Pita-Fuhui2663-KO-1* and *Pita-Fuhui2663-KO-2*) were generated using the CRISPR/Cas9 system and verified by sequencing. (**b**) Inoculation of rice blast fungus showed that the two knockout lines generated by CRISPR/Cas9 were susceptible to KJ201, while their parent Fuhui2663 was resistant to KJ201. Leaves were photographed 6 days post-infection with *M. oryzae* isolate KJ201. (**c**) Lesion numbers per cm^2^ on the rice leaves (M ± SD, n > 6 leaves) after inoculation with blast fungus as in (**b**). **denotes *P* < 0.01.

The original Article has been corrected.

