# Supplementary material for: Analysis of a rice blast resistance gene Pita-Fuhui2663 and development of selection marker
Source: Sci Rep. 2022 Sep 1;12:14917. doi: 10.1038/s41598-022-19004-y (PMC9437026; doi:10.1038/s41598-022-19004-y)

Supplementary Table 1 Primers used for synthesizing gRNA spacers and genotyping of CRISPR-edited mutants

Name	sequence 5'→3'	Purpose
gRNAs1- *Pita-Fuhui2663*	GTGTGGGATATTGTTAGCCG	CRISPR/CAS9
Pita-R-F	TGGCCAACAACTCCACTGAA	Screening of lines
Pita-R-Fuhui2663	TCAGCATGCTATCCCACGTA	Screening of lines
Pita-Fuhui2663-dCAPS-F	ATCACGGGTATGGATTTTTCA	Restriction enzyme
Pita-Fuhui2663-dCAPS-R	GTGCATCTTCAACCTGACTTGATGATTGTTTGA	Restriction enzyme

Supplementary Table 2. Indel and SSR molecular marker used for fine mapping the *Pita-R* gene

Marker	Sequence of forward primer	Sequence of reverse primer	Physical distance (kb)
Indel-12-4	ATCATTTCAGCCTGTGCC	AGCTTAATAGGGGGGACG	4203
RM27728	CCTCCATACTCACAAAGGAAGTCG	TCCCTCCGTACTTATAAAGGAAGTCG	5590
RM27766	ATGGTGCCCGAGTACAATGG	TGAGGTGCATGTGAACCTTGG	6438
RM27830	CTGTCCTGTGCCTCCTGTGC	GGTACGTCATCCGTGTATGAGTCG	7886
RM27860	AACCTTGGAGCAATATCACC	TGAATCACTAGCCGATAACC	8654
Indel-1	AGCATCCTAAGCACTTTAGC	TTGAGCATGTTGATCCTGT	9143
Indel-6	AACCAACTAGTCGCATATTA	TTCTTCCTTAACTCGTCGAA	9765
Indel-11	ACAACCAAGTTTGATCACTG	ATGCTACACACACGACTACG	10368
Indel-22	TGGGCAACTGAATCTAACCA	GGAGATGATGATGCGGTGAT	10474
Indel-26	CCCAAAGTCAGCTGAGAGTA	AGCCATACGACTGTAGGAAA	10572
Indel-30	TAGGAGGGGTATAATCGTCA	TTAGGGTAACATTGGCATTT	10622
Indel-33	TAACCTTCAGGCGTACTTTC	TTTTGCAAATTTTGATGTTG	10652
Indel-12	ATCCATTCCATTTGTACGAG	ATAGTTTTCACCACTTCCCA	10721
Indel-16	AAGCATGGATACAAACCATT	GCAAAAACAACCGTAAGAAG	11328
RM27960	CGGCCCGAACCTGTATAACC	AGGAGTATCCTTTCTCGCAATCG	11935
RM27983	GCAAGCGTGGGATTCTCTCC	CTATGCCAGATGGTGCTTACTCG	12685
Indel-12-7	CAACTAAAACCAACACAAAATCCA	TGTCTAGTTGCATGTCTGAGTGTC	13198

Supplementary Table 3. Mutation sites of two target mutant lines

Line	Target type	Mutation site
Pita-Fuhui2663-KO-1	gRNAs 1	TATGGGCTTCATCAATGCTGGGATATTGTTAGCCGTGGTTTGCC (1 bp insertion)
Pita-Fuhui2663-KO-2	gRNAs 1	TATGGGCTTCATCAATG - GGGATATTGTTAGCCGTGGTTTGCC (1 bp deletion)

Supplementary Table 4. Haplotype analysis of *Pita-Fuhui2663*

Haplotype	SNP	Sample list	Group	Plant Height (2799)
Hap1	TCCACTGCCCCA	680	Aus: 2; Bas: 12; GJ: 13; XI: 645; admix: 8	(617 of 680) mean: 96.408
Hap2	TACACTGCGCCC	651	Aus: 82; Bas: 15; GJ: 37; XI: 504; admix: 13	(584 of 651) mean: 96.731
Hap3	CACTCTGCGTCC	359	Aus: 5; GJ: 203; XI: 137; admix: 13; na: 1	(333 of 359) mean: 99.700
Hap4	TACACTGCGCCA	349	Aus: 106; Bas: 29; GJ: 5; XI: 195; admix: 14	(338 of 349) mean: 90.322
Hap5	TACACACGGCCC	299	Bas: 6; GJ: 282; XI: 3; admix: 8	(279 of 299) mean: 99.315
Hap6	TATACACGGCCC	254	GJ: 239; XI: 8; admix: 7	(246 of 254) mean: 85.077
Hap7	TACAATGCGCCC	158	Bas: 6; XI: 147; admix: 5	(149 of 158) mean: 92.970
Hap8	TACACACGGCAC	31	GJ: 20; XI: 11	(31 of 31) mean: 100.935
Hap9	TMCACTGCSCCM	23	XI: 19; admix: 4	(20 of 23) mean: 94.950
Hap10	YMCWCTGCSYCM	8	XI: 7; admix: 1	(8 of 8) mean: 97.938
Hap11	TAYACACGGCCC	7	GJ: 7	(6 of 7) mean: 87.000
Hap12	TMCACTGCCCCA	7	XI: 7	(6 of 7) mean: 87.333
Hap13	TACACWSSGCCC	6	XI: 3; admix: 3	(4 of 6) mean: 98.500
Hap14	TACAMTGCGCCC	6	XI: 6	(6 of 6) mean: 99.000
Hap15	TACACTGCGCCM	6	Aus: 1; Bas: 1; XI: 3; admix: 1	(4 of 6) mean: 89.250
Hap16	TAYACWSSGCCC	5	GJ: 1; XI: 2; admix: 2	(5 of 5) mean: 95.200
Hap17	TMCAMTGCSCCM	4	XI: 4	(4 of 4) mean: 110.750
Hap18	TMCACTGCSCCA	4	XI: 4	(4 of 4) mean: 89.250
Hap19	YACWCTGCGYCM	4	admix: 4	(4 of 4) mean: 95.500
Hap20	YACWCWSSGYCC	4	GJ: 4	(4 of 4) mean: 110.625
Hap21	TMCACWSSSCCM	3	GJ: 1; XI: 1; admix: 1	(2 of 3) mean: 102.500
Hap22	TACACASSGCCC	3	Bas: 1; GJ: 1; admix: 1	(3 of 3) mean: 105.667
Hap23	TACACWSSGCCM	3	XI: 1; admix: 2	(3 of 3) mean: 90.667
Hap24	YACWMTGCGYCC	3	XI: 1; admix: 2	(2 of 3) mean: 86.000
Hap25	TMCACTGCGCCM	3	XI: 2; admix: 1	(3 of 3) mean: 97.167

Note: XI means Xian/indica subpopulation; GJ means Geng/japonica types; Aus means *centrum*-Aus population; Bas means *centrum*-Basmati population; admix means admixed between any two or more of the XI, GJ, Aus, Bas populations.

Supplementary Figure 1. Fuhui2663 is resistant to KJ201 and LTH is susceptible to KJ201 under laboratory conditions.

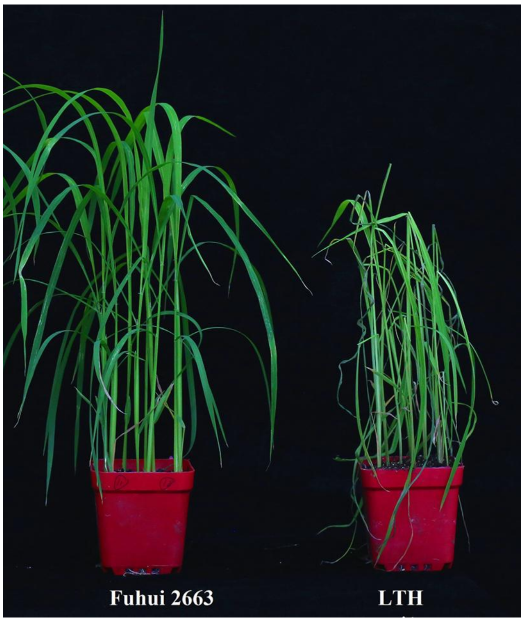

Supplementary Figure 2. *Ptr* was located about 210 kb upstream of *Pita-Fuhui2663* and *Pita*

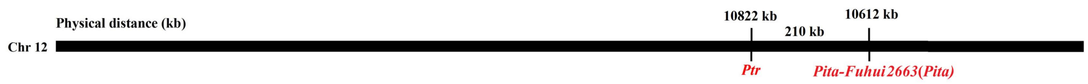

Supplementary Figure 3. Amino acid sequence comparison among Pita-Fuhui2663-KO-1, Pita-Fuhui2663-KO-1, Pita-Fuhui2663 and Pita-S

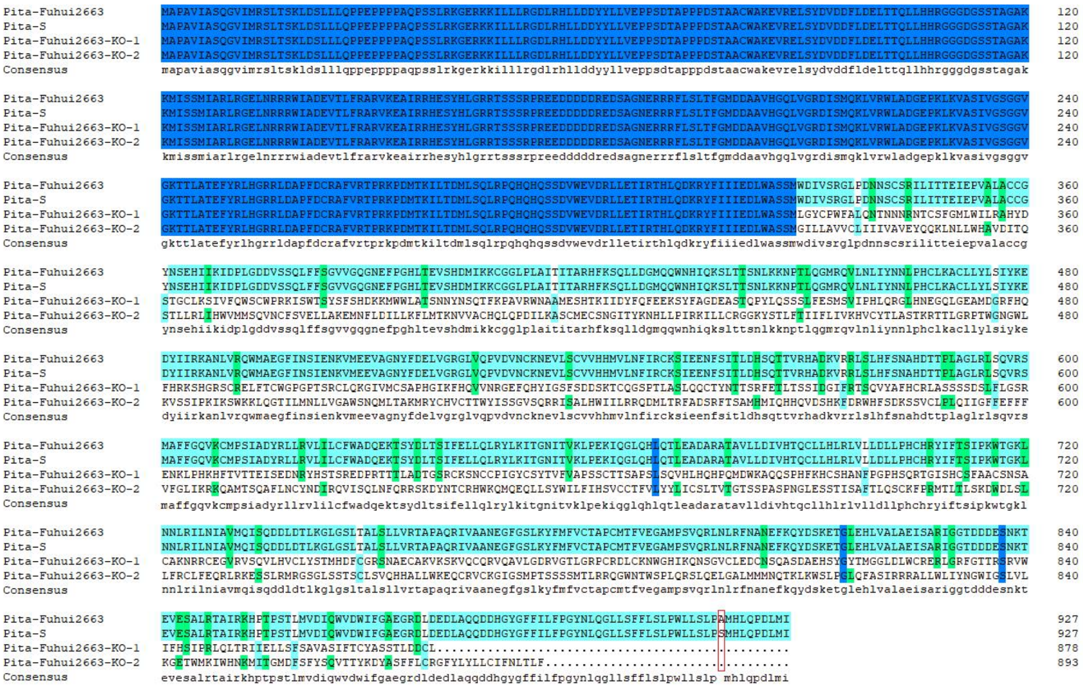

Supplementary Figure 4. Detection and analysis of *Pita-Fuhui2663-dCAPS* molecular marker. M represents DNA size markers; Lane 1 shows the amplified target fragment of 123 bp from *Pita-Fuhui2663*; Lane 2 shows the amplified target fragment of 98 bp from *Pita-S*.

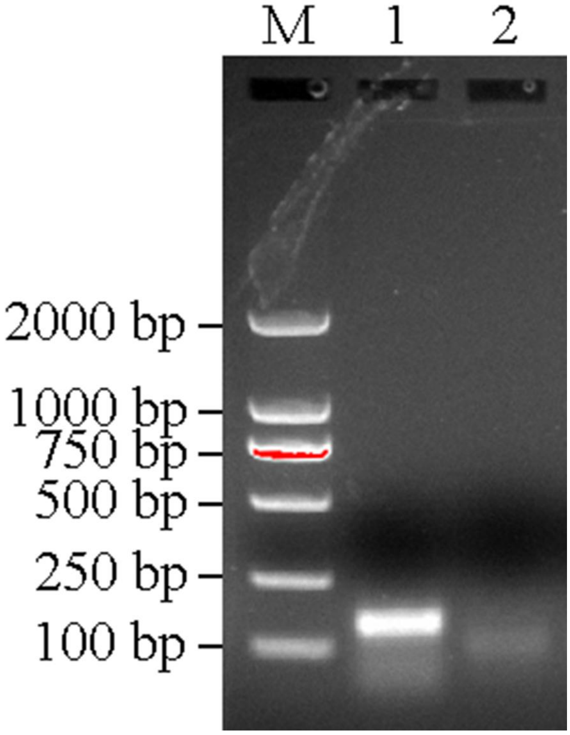

Supplement: Supplementary file 1 — Supplementary Information. [file 41598_2022_19004_MOESM1_ESM.pdf]
